# Comparison of different definitions of pathologic complete response in operable breast cancer: a pooled analysis of three prospective neoadjuvant studies of JBCRG

**DOI:** 10.1007/s12282-014-0524-4

**Published:** 2014-02-27

**Authors:** Katsumasa Kuroi, Masakazu Toi, Shinji Ohno, Seigo Nakamura, Hiroji Iwata, Norikazu Masuda, Nobuaki Sato, Hitoshi Tsuda, Masafumi Kurosumi, Futoshi Akiyama

**Affiliations:** 1 Department of Surgery, Tokyo Metropolitan Cancer and Infectious Diseases Center Komagome Hospital, 3-18-22 Honkomagome, Bunkyo-ku, Tokyo, 113-8677 Japan; 2 Department of Surgery (Breast Surgery), Graduate School of Medicine Kyoto University, Kyoto, Japan; 3 Department of Clinical Oncology, The Clinical Institute of NHO Kyusyu Cancer Center, Fukuoka, Japan; 4 Division of Breast Surgical Oncology, Department of Surgery, Showa University School of Medicine, Tokyo, Japan; 5 Department of Breast Surgical Oncology, St. Luke’s International Hospital, Tokyo, Japan; 6 Department of Breast Oncology, Aichi Cancer Center Hospital, Aichi, Japan; 7 Department of Surgery, Breast Oncology, NHO Osaka National Hospital, Osaka, Japan; 8 Department of Breast Oncology, Niigata Cancer Center Hospital, Niigata, Japan; 9 Department of Basic Pathology, National Defense Medical College, Saitama, Japan; 10 Department of Pathology, Saitama Cancer Center, Saitama, Japan; 11 Department of Pathology, The Cancer Institute of Japanese Foundation for Cancer Research, Tokyo, Japan

**Keywords:** Neoadjuvant chemotherapy, Pathologic response, Subtype, Breast cancer

## Abstract

**Background:**

Neoadjuvant chemotherapy (NAC) has been accepted as one of the standard treatments for operable breast cancer. However, the term pathologic complete response (pCR) has not been consistently defined.

**Methods:**

This study was a pooled analysis of three prospective studies of NAC conducted by JBCRG and was performed to compare the prognostic significance of different definitions of pCR. pCRs were defined as follows: QpCR, few or no remaining invasive cancer cells in the breast; CpCR, ypT0/is; CpCRbn, ypT0/isypN0; SpCR, ypT0; SpCRbn, ypT0ypN0; Grade 2b, only a few remaining cancer cells in the breast.

**Results:**

A total of 353 patients were included. A Cox proportional hazards model revealed that hazard ratios (HRs) of each pCR were lower than 1; however, pCR was significant for disease-free survival (DFS) and overall survival (OS) only when QpCR, CpCR, and CpCRbn were used (DFS; QpCR, 0.27; CpCR, 0.39; CpCRbn, 0.42, SpCR, 0.57, SpCRbn, 0.68: OS; QpCR, 0.12; CpCR, 0.17; CpCRbn, 0.16; SpCR, 0.30, SpCRbn, 0.45). Grade 2b was also a significant prognostic variable for DFS and OS (HR: DFS, 0.19; OS, 0.15). Neither bone nor brain was the first site of recurrence in patients who achieved pCR, irrespective of the definition of pCR. Triple-negative and Her2-positive tumors tended to recur in soft tissue more frequently than the other subtypes, and luminal tumors had the lowest rate of recurrence in the brain.

**Conclusion:**

Prognostic significance of pCR varied according to the definition of pCR, and the pattern of recurrence might be different according to pathologic response and subtype.

## Introduction

Neoadjuvant chemotherapy (NAC) has been accepted as one of the standard treatments for operable breast cancer. The prognosis of patients treated with NAC is at least equivalent to the prognosis of patients treated with postoperative adjuvant chemotherapy; NAC improves surgical options through tumor shrinkage, and is useful for testing the treatment response [1, 2]. Patients with a pathologic complete response (pCR) have a better prognosis than patients who did not achieve a pCR [1, 2]. However, as several definitions of pCR have been used, the term pCR has not been applied in a consistent manner [3, 4]. According to some definition, the presence of an intraductal component is negligible, or invasive residual disease is acceptable if minimal, while others require that there must be no histologic evidence of residual cancer cells in the breast and axillary lymph nodes (LNs) [1, 3–7]. Under these conditions, FDA has proposed the use of ypT0/isypN0 as an endpoint to support accelerated approval regulations in 2012 [8].

According to the histological response criteria of the Japanese Breast Cancer Society (JBCS), pathologic response was categorized into 6 grades (Grade 0, 1a, 1b, 2a, 2b, 3) based on histological change in the invasive area, and in the past decade the Japan Breast Cancer Research Group (JBCRG) has conducted three prospective phase II studies of NAC, JBCRG-01, JBCRG-02 and JBCRG-03, which have examined sequential combinations of fluorouracil, epirubicin and cyclophosphamide (FEC), and docetaxel [3, 9–12]. In these studies, the invasive component, intraductal component, and LN metastasis were individually evaluated, and we could apply several definitions of pCR to the same patient. The present study was a pooled analysis of these JBCRG studies performed to compare the prognostic significance of several different definitions of pCR.

## Patients and methods

### JBCRG studies of NAC

Details of JBCRG-01, JBCRG-02, and JBCRG-03 studies have been described previously [10–12]. In brief, the three studies had comparable main eligibility criteria. The diagnosis of invasive breast cancer was histologically confirmed in all patients by core biopsy. Female patients needed to have a measurable breast tumor of at least 1 cm in diameter. Locally advanced or inflammatory breast cancer was not eligible. Prior to surgery, 4 cycles of fluorouracil (500 mg/m^2^), epirubicin (100 mg/m^2^), and cyclophosphamide (500 mg/m^2^), q3w followed by 4 cycles of DOC (75 mg/m^2^), q3w were administered in JBCRG-01, and the dose of DOC was increased to 100 mg/m^2^ in JBCRG-02 [10, 11]. In JBCRG-03, FEC and DOC were administered in reverse order from JBCRG-01 [12]. Patients with hormone receptor (HR)-positive tumors were encouraged to receive adjuvant endocrine treatment for at least 5 years, and adjuvant radiation therapy was recommended for patients who underwent breast-conserving surgery. No patients received trastuzumab as a part of NAC; however, after the approval of adjuvant use of trastuzumab in 2008, patients could receive trastuzumab for 1 year, if indicated. All studies were approved by the relevant ethics committees, and all patients provided written informed consent for study participation and data collection. All studies were registered to UMIN (JBCRG-01, C000000011; JBCRG-02, C000000020, C000000320; JBCRG-03, C000000291).

### Patients

For this pooled analysis, individual patient data regarding baseline characteristics, histopathological results at diagnosis and surgery, and follow-up were extracted from the original databases. Only patients who received at least one cycle of systemic chemotherapy were included. Patients were excluded due to missing data for ER, PgR, Her2, or surgery and due to ineligibility or withdrawal of consent. Finally, among 389 patients who were enrolled in JBCRG-01, JBCRG-02, and JBCRG-03, 353 patients were included in the present study. The detailed patients’ characteristics have been summarized in the previous articles [13, 14]. In brief, 200 patients received adjuvant endocrine therapy according to protocol and practice guidelines, and after the approval of trastuzumab for adjuvant use, 17 patients received postoperative trastuzumab for 1 year. Ki-67 was not available for the majority of patients, and nuclear grade was not assessed in 106 patients (30.0 %).

### Assessment of response

Clinical tumor assessments were performed at each institute within 4 weeks before initiation of NAC, after completion of the first 4 cycles of chemotherapy, and before surgery according to the modified Response Evaluation Criteria in Solid Tumors (RECIST) guidelines. Clinical examinations were based on palpable changes in tumor size in combination with mammography, ultrasonography, computed tomography (CT), and magnetic resonance imaging (MRI).

Pathologic response was independently evaluated by a blinded central review committee according to the JBCS criteria [3, 9]. For an assessment of pCR, multiple tumor sections were examined, and cytokeratin immunostaining was performed to confirm the presence of residual cancer cells (RDs), if required. pCR was defined as follows: quasi pCR (QpCR), no invasive RD in the breast, but noninvasive RDs, only a few remaining invasive RDs and infiltrated LNs allowed; comprehensive pCR (CpCR), no invasive RD in the breast but noninvasive breast RDs and infiltrated LNs allowed, i.e., ypT0/is or Grade 3; CpCRbn, no invasive RD in the breast and LNs but noninvasive breast RDs allowed, i.e., ypT0/isypN0; strict pCR (SpCR), no invasive and noninvasive RD in the breast, i.e., ypT0; and SpCRbn, no invasive and noninvasive RD in the breast and LNs, i.e., ypT0ypN0. Furthermore, we defined three categories of RD as follows: pCRinv, only noninvasive breast RDs in the breast, i.e., ypTis; Grade 2b, marked changes approaching a complete response with only a few RDs in the breast; and Grade 0–2a, no or slight response, or marked changes in two-thirds or more of tumor cells with apparent RDs in the breast.

### Assessment of HR and Her2

Estrogen receptor (ER) status and progesterone receptor (PgR) status were determined by immunohistochemistry at each institute, and in general, tumors with >10 % positively stained tumor cells were classified as positive for ER and PgR. Her2 status was also determined at each institute by immunohistochemistry or by fluorescence in situ hybridization (FISH) analysis. Her2-positive tumors were defined as 3+ on immunohistochemistry or as positive by FISH. Subtypes were classified into luminal tumors (ER-positive and/or PgR-positive, Her2-negative), luminal/Her2-positive tumors (ER-positive and/or PgR-positive, Her2-positive), Her2-positive tumors (ER-negative, PgR-negative, Her2-positive), and triple-negative (TN) tumors (ER-negative, PgR-negative, Her2-negative).

### Statistical analysis

Comparisons between groups were made with the Chi-square test or Fisher’s exact test for proportions and Wilcoxon test for continuous variables. The Kaplan–Meier methods were used to calculate disease-free survival (DFS) and overall survival (OS) from the date of initiation of NAC to the date of last follow-up, recurrence, secondary cancers, contralateral breast cancers, or death. Comparisons were made using the log-rank test. Hazard ratios (HRs), 95 % confidence interval (CI), and corresponding *p* values were calculated using the Cox proportional hazards model. In multivariate analysis, variables were chosen on the basis of goodness of fit. Statistical analyses were performed with JMP (version 10, SAS Institute Inc.), and *p* < 0.05 was considered statistically significant.

## Results

The rates of QpCR, CpCR, CpCRbn, SpCR, and SpCRbn were 27.8, 20.4, 18.4, 9.9, and 8.2 %, respectively (Table 1). Luminal/Her2-positive, Her2-positive and TN tumors showed significantly higher pCR rates than luminal tumors (*p* < 0.001) irrespective of the definition of pCR. Nuclear grade, nodal status, and clinical response were also associated with pCRs (*p* < 0.05), although there was no significant association between QpCR and clinical response before surgery (*p* = 0.06).

**Table 1:** Patient characteristics and corresponding pCR rates

	All patients	QpCR		CpCR		CpCRbn		SpCR		SpCRbn	*p* value
All patients	353 (100)	98 (27.8 %)		72 (20.4)		65 (18.4)		35 (9.9 %)		29 (8.2 %)	
Study											
JBCRG-01	186 (52.7)	47 (25.3)	*p* = 0.43	31 (16.7)	*p* = 0.13	29 (15.6)	*p* = 0.32	15 (8.1)	*p* = 0.32	14 (7.5)	*p* = 0.81
JBCRG-02	37 (10.5)	13 (35.1)		11 (29.7)		9 (24.3)		6 (16.2)		4 (10.8)	
JBCRG-03	130 (36.8)	38 (29.3)		30 (23.1)		27 (20.8)		14 (10.8)		11 (8.5)	
Age											
<50	231 (65.4)	61 (26.4)	*p* = 0.44	45 (19.5)	*p* = 0.56	39 (16.9)	*p* = 0.31	26 (11.3)	*p* = 0.24	21 (9.1)	*p* = 0.40
≥50	122 (34.6)	37 (30.3)		27 (22.1)		26 (21.3)		9 (7.4)		8 (6.6)	
Tumor											
≤3 cm	207 (58.6)	55 (26.6)	*p* = 0.56	42 (20.3)	*p* = 0.95	36 (17.4)	*p* = 0.56	20 (9.7)	*p* = 0.85	15 (7.3)	*p* = 0.43
>3 cm	146 (41.4)	43 (29.5)		30 (20.6)		29 (19.9)		15 (10.3)		14 (9.6)	
Nuclear grade											
Grade 1	69 (19.6)	11 (15.9)	*p* = 0.036	5 (7.3)	*p* = 0.004	5 (7.3)	*p* = 0.009	2 (2.9)	*p* = 0.011	2 (2.9)	*p* = 0.016
Grade 2	102 (28.9)	31 (30.4)		24 (23.5)		21 (20.6)		9 (8.8)		7 (6.9)	
Grade 3	76 (21.5)	25 (32.9)		20 (26.3)		19 (25.0)		13 (17.1)		12 (15.8)	
Unknown	106 (30.0)										
Nodal status											
n0	143 (40.5)	83 (39.5)	*p* < 0.001	65 (31.0)	*p* < 0.001	65 (31.0)	*p* < 0.001	29 (13.8)	*p* = 0.002	29 (13.8)	*p* < 0.001
n+	210 (59.5)	15 (10.5)		7 (4.9)		0 (0.0)		6 (4.2)		0 (0.0)	
Subtype											
Luminal	206 (58.4)	32 (15.5)	*p* < 0.001	19 (9.2)	*p* < 0.001	18 (8.7)	*p* < 0.001	6 (2.9)	*p* < 0.001	5 (2.4)	*p* < 0.001
Luminal/Her2-positive	34 (9.6)	14 (41.2)		8 (23.5)		7 (20.6)		5 (14.7)		4 (11.8)	
Her2-positive	40 (11.3)	21 (52.5)		17 (42.5)		15 (37.5)		6 (15.0)		5 (12.5)	
Triple negative	73 (20.7)	31 (42.5)		28 (38.4)		25 (34.3)		18 (24.7)		15 (20.6)	
Clinical response after the first half of NAC											
CR, PR	214 (60.6)	69 (32.2)	*p* = 0.018	51 (23.8)	*p* = 0.044	47 (22.0)	*p* = 0.030	27 (12.6)	*p* = 0.029	23 (10.8)	*p* = 0.025
SD, PD	139 (39.4)	29 (20.9)		21 (15.1)		18 (13.0)		8 (5.8)		6 (4.3)	
Clinical response before surgery											
CR, PR	261 (74.0)	82 (31.4)	*p* = 0.06	62 (23.8)	*p* = 0.003	56 (21.5)	*p* = 0.005	31 (11.9)	*p* = 0.031	26 (10.0)	*p* = 0.034
SD, PD	89 (25.2)	15 (16.9)		9 (10.1)		8 (9.0)		4 (4.5)		3 (3.4)	
Unknown	3 (0.8)										

*CR* complete response, *NAC* neoadjuvant chemotherapy, *n+* node positive, *PR* partial response, *SD* stable disease, *PD* progressive disease

**Fig. 1:**
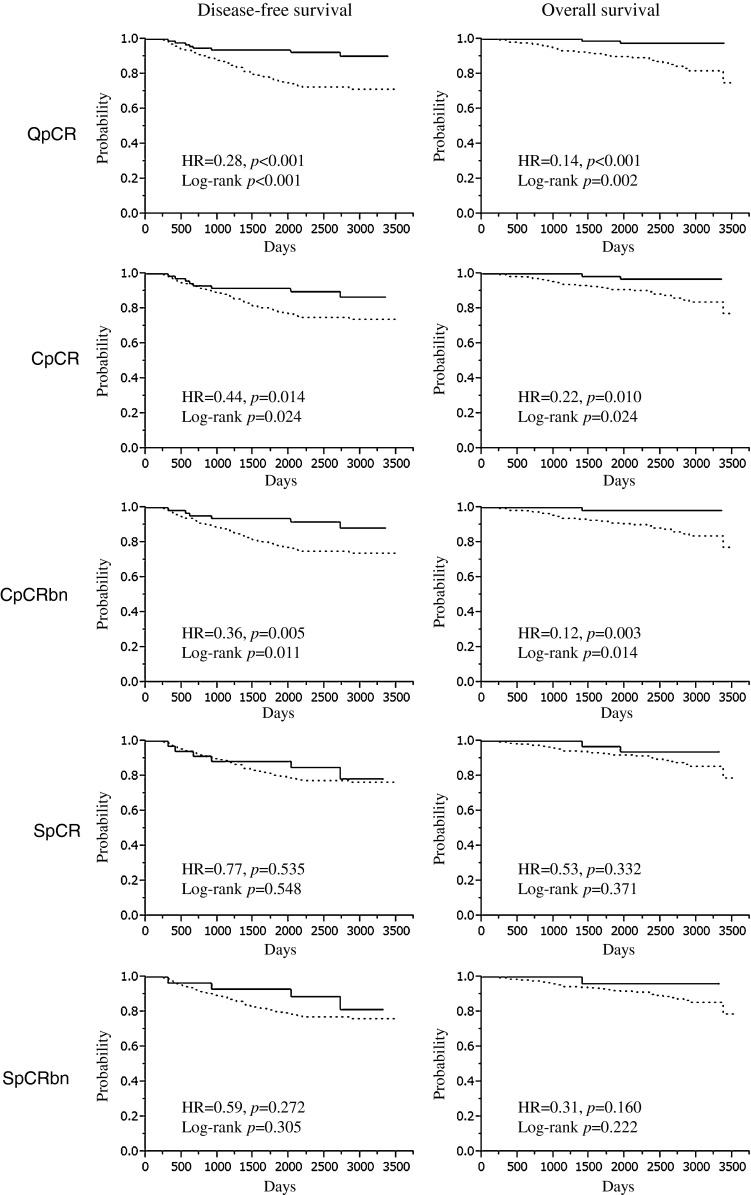
Association between various definition of pathologic complete response and survival

With a median follow-up of 2,274 days, patients who achieved pCR had significantly improved DFS as compared to patients without pCR when QpCR, CpCR, and CpCRbn were used, while there were no significant differences between pCR and DFS in SpCR and SpCRbn (QpCR, log-rank, *p* < 0.001, HR = 0.28, *p* < 0.001; CpCR, log-rank, *p* = 0.024, HR = 0.44, *p* = 0.014; CpCRbn, log-rank, *p* = 0.011, HR = 0.36, *p* = 0.005; SpCR, log-rank, *p* = 0.548, HR = 0.77, *p* = 0.535; SpCRbn, log-rank, *p* = 0.305: HR = 0.59, *p* = 0.272) (Fig. 1). For OS, similar results were observed (QpCR, log-rank, *p* = 0.002, HR = 0.14, *p* < 0.001; CpCR, log-rank, *p* = 0.024, HR = 0.22, *p* = 0.010; CpCRbn, log-rank, *p* = 0.014, HR = 0.12, *p* = 0.003; SpCR, log-rank, *p* = 0.371, HR = 0.53, *p* = 0.332; SpCRbn, log-rank, *p* = 0.222, HR = 0.31, *p* = 0.160). A Cox proportional hazards model that included pCR, study, age, tumor size, nuclear grade, nodal status, subtype, and clinical response found that prognostic significance of nodal status (n + vs n0) and subtype (TN vs luminal) were consistent irrespective of the definition of pCR for DFS and OS (*p* < 0.01) (Table 2). HRs of each pCR were lower than 1; however, it was significant for DFS and OS only when QpCR, CpCR or CpCRbn was used as the definition of pCR (DFS; QpCR, *p* < 0.01; CpCR, *p* < 0.05; CpCRbn, *p* < 0.05: OS; QpCR, *p* < 0.01; CpCR, *p* < 0.05; CpCRbn, *p* < 0.05). Tumor size was the significant prognostic variable for OS when CpCR, CpCRbn, SpCR or SpCRbn was used as the definition of pCR (CpCR, *p* < 0.05; CpCRbn, *p* < 0.05; SpCR, *p* < 0.05; SpCRbn, *p* < 0.05).

**Table 2:** Prognostic impact of pCR on survival (Cox proportional hazards model)

Variables	QpCR		CpCR		CpCRbn		SpCR		SpCRbn	
	HR	95 % CI	HR	95 % CI	HR	95 % CI	HR	95 % CI	HR	95 % CI
Disease-free survival										
Study										
JBCRG-02	2.09	0.95–4.25	1.96	0.89–3.98	1.66	0.76–3.33	1.87	0.84–3.83	1.67	0.77–3.34
JBCRG-03	1.31	0.76–2.21	1.29	0.75–2.17	1.26	0.74–2.13	1.25	0.73–2.12	1.22	0.72–2.07
Pathologic response										
pCR	0.27**	0.11–0.56	0.39*	0.16–0.85	0.42*	0.15–0.99	0.57	0.21–1.34	0.68	0.20–1.80
Age	1.00	0.97–1.03	1.00	0.97–1.03	1.00	0.97–1.03	1.00	0.97–1.03	1.00	0.97–1.03
Tumor size										
>3 cm	1.19	0.73–1.98	1.24	0.76–2.05	1.25	0.77–2.08	1.27	0.78–2.11	1.29	0.79–2.14
Nuclear grade										
Grade 3	1.31	0.66–2.55	1.38	0.70–2.66	1.43	0.73–2.75	1.53	0.79–2.92	1.54	0.80–2.94
Nodal status										
n+	2.29**	1.40–3.81	2.45**	1.49–4.08	2.27**	1.36–3.87	2.80**	1.71–4.62	2.70**	1.64–4.53
Clinical response (CR, PR)										
After the first half of NAC	0.74	0.44–1.27	0.71	0.42–1.21	0.73	0.43–1.24	0.73	0.43–1.23	0.73	0.44–1.25
Before surgery	0.88	0.48–1.50	0.85	0.48–1.52	0.85	0.48–1.53	0.82	0.47–1.47	0.82	0.47–1.48
Subtype										
Luminal/Her2-positive	1.62	0.60–3.73	1.37	0.51–3.11	1.28	0.48–2.87	1.32	0.49–3.00	1.26	0.47–2.85
Her2-positive	1.33	0.48–3.11	1.15	0.42–2.68	1.03	0.38–2.37	0.97	0.35–2.25	0.91	0.33–2.11
Triple negative	3.39**	1.82–6.19	3.25**	1.73–5.96	2.88**	1.56–5.18	2.89**	1.55–5.29	2.66**	1.45–4.77
Overall survival										
Study										
JBCRG-02	2.85	0.92–7.81	2.69	0.87–7.38	1.87	0.62–4.98	2.56	0.82–7.06	1.92	0.64–5.02
JBCRG-03	1.42	0.57–3.42	1.44	0.59–3.44	1.38	0.57–3.28	1.41	0.58–3.34	1.33	0.55–3.13
Pathologic response										
pCR	0.12**	0.02–0.43	0.17*	0.03–0.62	0.16*	0.01–0.84	0.30	0.04–1.18	0.45	0.02–2.43
Age	0.98	0.94–1.03	0.98	0.94–1.03	0.98	0.94–1.03	0.98	0.94–1.02	0.98	0.94–1.02
Tumor size										
>3 cm	2.03	0.98–4.54	2.14*	1.03–4.80	2.16*	1.03–4.86	2.22*	1.07–4.95	2.25*	1.08–5.07
Nuclear grade										
Grade 3	1.07	0.39–2.81	1.14	0.42–2.97	1.17	0.44–3.03	1.31	0.49–3.33	1.30	0.49–3.31
Nodal status										
n+	3.05**	1.47–6.63	3.18**	1.54–6.91	2.69**	1.29–5.96	3.85**	1.86–8.33	3.49**	1.68–7.72
Clinical response (CR, PR)										
After the first half of NAC	0.76	0.33–1.71	0.70	0.31–1.56	0.71	0.32–1.57	0.72	0.33–1.59	0.71	0.32–1.57
Before surgery	0.55	0.25–1.26	0.58	0.26–1.32	0.54	0.24–1.23	0.53	0.24–1.20	0.51	0.22–1.15
Subtype										
Luminal/Her2-positive	2.73	0.60–9.08	2.14	0.48–6.85	1.84	0.42–5.81	2.04	0.46–6.56	1.83	0.41–5.80
Her2-positive	3.31	0.88–10.19	2.79	0.74–8.53	2.28	0.61–6.83	2.17	0.58–6.53	1.93	0.52–5.82
Triple negative	4.92**	2.07–11.42	4.85**	2.04–11.29	2.94**	1.67–9.07	4.23**	1.78–9.78	3.59**	1.54–8.17

* *p* < 0.05, ** *p* < 0.01

*CI* confidence interval, *CR* complete response, *HR* hazard ratio, *n+* node positive, *PR* partial response

**Fig. 2:**
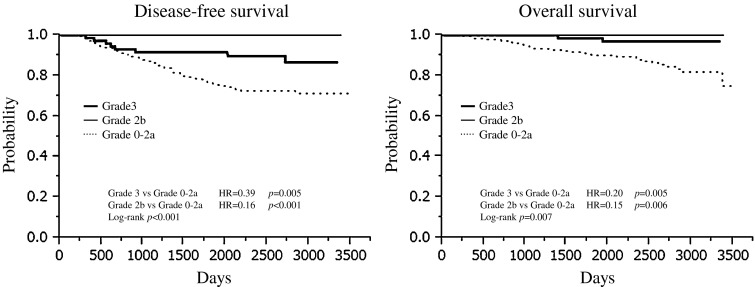
Survival according to pathologic response defined by the classification of Japanese Breast Cancer Society

As shown in Table 3, the rates of SpCR, pCRinv, Grade 2b, and Grade 0–2a were 9.9, 10.5, 7.4, and 72.2 %, respectively, and univariate analysis showed significant association between pathologic response and nuclear grade, nodal status, subtype, and clinical response before surgery (nuclear grade, *p* = 0.028; nodal status*, p* < 0.001, subtype, *p* < 0.001, clinical response before surgery, *p* = 0.028). Patients who achieved Grade 3 or Grade 2b experienced longer DFS and OS than those with Grade 0–2a (DFS; log-rank, *p* < 0.001; Grade 3, HR = 0.39, *p* = 0.005; Grade 2b, HR = 0.16, *p* < 0.001: OS; log-rank, *p* = 0.007; Grade 3, HR = 0.20, *p* = 0.005; Grade 2b, HR = 0.15, *p* = 0.006) (Fig. 2). A Cox proportional hazards model found that pathologic response (Grade 3, Grade 2b vs Grade 0–2a), nodal status (n + vs n0), and subtype (TN vs luminal) were significant prognostic variables for DFS and OS (DFS; Grade 3, HR = 0.5, *p* < 0.001; Grade 2b, HR = 0.19, *p* < 0.001; n+, HR = 2.33, *p* < 0.001, TN, HR = 3.19, *p* < 0.001: OS; Grade 3, HR = 0.15, *p* < 0.001; Grade 2b, HR = 0.15, *p* < 0.001; n+, HR = 3.06, *p* < 0.001, TN, HR = 4.80, *p* < 0.001) (Table 4).

**Table 3:** Association between patient characteristics and pathologic response defined by the classification of Japanese Breast Cancer Society

Variables	QpCR				
	Grade 3		Grade 2b	Grade 0–2a	*p* value
	SpCR	pCRinv			
All patients	35 (9.9)	37 (10.5)	26 (7.4)	255 (72.2)	
Study					
JBCRG-01	15 (8.1)	16 (8.6)	16 (8.6)	139 (74.7)	0.58
JBCRG-02	6 (16.2)	5 (13.5)	2 (5.4)	24 (64.9)	
JBCRG-03	14 (10.8)	16 (12.3)	8 (6.2)	92 (70.8)	
Age					
<50	26 (11.3)	19 (8.2)	16 (6.9)	170 (73.6)	0.19
≥50	9 (7.4)	18 (14.8)	10 (8.2)	85 (69.7)	
Tumor					
≤3 cm	20 (9.7)	22 (10.6)	13 (6.3)	152 (73.4)	0.82
>3 cm	15 (10.3)	15 (10.3)	13 (8.9)	103 (70.6)	
Nuclear grade					
Grade 1	2 (2.9)	3 (4.4)	6 (8.7)	58 (84.1)	0.028
Grade 2	9 (8.8)	15 (14.7)	7 (6.9)	71 (69.6)	
Grade 3	13 (17.1)	7 (9.2)	5 (6.6)	51 (67.1)	
Nodal status					
n0	29 (13.8)	36 (17.1)	18 (8.6)	127 (60.5)	<0.001
n+	6 (4.2)	1 (0.7)	8 (5.6)	128 (89.5)	
Subtype					
Luminal	6 (2.9)	13 (6.3)	13 (6.3)	174 (84.5)	<0.001
Luminal/Her2-positive	5 (14.7)	3 (8.8)	6 (17.7)	20 (58.8)	
Her2-positive	6 (15.0)	1 (27.5)	4 (10.0)	19 (47.5)	
Triple negative	18 (24.7)	10 (13.7)	3 (4.1)	42 (57.5)	
Clinical response after the first half of NAC					
CR, PR	27 (12.6)	24 (11.2)	18 (8.4)	145 (67.8)	0.07
SD, PD	8 (5.8)	13 (9.4)	8 (5.8)	110 (79.1)	
Clinical response before surgery					
CR, PR	31 (11.9)	31 (11.9)	20 (7.7)	179 (68.6)	0.028
SD, PD	4 (4.5)	5 (5.6)	6 (6.7)	74 (83.2)	

*CR* complete response, *NAC* neoadjuvant chemotherapy, *n+* node positive, *PR* partial response, *SD* stable disease, *PD* progressive disease

**Table 4:** Prognostic impact of Grade 3 and Grade 2b on survival (Cox proportional hazards model)

Variables	Disease-free survival		Overall survival	
	HR	95 % CI	HR	95 % CI
Study				
JBCRG-02	2.02	0.92–4.11	2.80	0.91–7.64
JBCRG-03	1.29	0.75–2.18	1.40	0.57–3.38
Pathologic response				
Grade 3	0.35**	0.15–0.75	0.15**	0.02–0.55
Grade 2b	0.19**	0–0.32	0.15*	0–0.68
Age	1.00	0.97–1.03	0.98	0.94–1.03
Tumor size				
>3 cm	1.20	0.74–1.99	2.03	0.98–4.54
Nuclear grade				
Grade 3	1.34	0.68–2.60	1.07	0.39–2.81
Nodal status				
n+	2.33**	1.41–3.89	3.06**	1.48–6.67
Clinical response (CR, PR)				
After the first half of NAC	0.77	0.45–1.30	0.77	0.34–1.74
Before surgery	0.83	0.47–1.48	0.54	0.24–1.23
Subtype				
Luminal-Her2-positive	1.68	0.62–3.87	2.81	0.62–9.34
Her2-positive	1.31	0.47–3.07	3.32	0.88–10.24
Triple negative	3.19**	1.70–5.86	4.80**	2.02–11.18

* *p* < 0.05, ** *p* < 0.01

*CI* confidence interval, *CR* complete response, *HR* hazard ratio, *NAC* neoadjuvant chemotherapy, *PR* partial response, *SD* stable disease, *PD* progressive disease

When the first sites of recurrence were analyzed according to pCR and subtype, neither bone nor brain was the first site of recurrence in patients with pCR, irrespective of the definition of pCR (Table 5). In patients who achieved Grade 2b, no recurrence was observed. On the other hand, bone was not the first site of recurrence in patients with luminal/Her2-positive and Her2-positive tumors, and soft tissue recurrence was not observed in patients with luminal/Her2-positive tumors. Her2-positive or TN tumors tended to recur in soft tissue more frequently than the other subtypes, and luminal tumors had a lower rate of recurrence in brain. Viscera were the most common sites of first recurrence independent of the definition of pCR and subtype.

**Table 5:** First site of recurrence in terms of pCR and subtype

Category	First site of recurrence (%)				*p* value
	Soft tissue	Bone	Viscera	Brain	
QpCR					
pCR	3 (50.0)	0	3 (50.0)	0	0.26
Non-pCR	17 (27.9)	9 (14.8)	27 (44.2)	8 (13.1)	
CpCR					
pCR	3 (50.0)	0	3 (50.0)	0	0.26
Non-pCR	17 (27.9)	9 (14.8)	27 (44.2)	8 (13.1)	
CpCRbn					
pCR	2 (40.0)	0	3 (60.0)	0	0.38
Non-pCR	18 (29.0)	9 (14.5)	27 (43.6)	8 (12.9)	
SpCR					
pCR	2 (50.0)	0	2 (50.0)	0	0.46
Non-pCR	18 (28.6)	9 (14.3)	28 (44.4)	8 (12.7)	
SpCRbn					
pCR	1 (33.3)	0	2 (66.7)	0	0.60
Non-pCR	19 (29.7)	9 (14.0)	28 (43.8)	8 (12.5)	
JBCS					
Grade 3	3 (50.0)	0	3 (50.0)	0	0.26
Grade 2b	0	0	0	0	
Grade 0–2a	17 (27.9)	9 (14.8)	27 (44.2)	8 (13.1)	
Subtype					
Luminal	10 (27.8)	7 (19.4)	17 (47.2)	2 (5.6)	0.13
Luminal/Her2-positive	0	0	5 (83.3)	1 (16.7)	
Her2-positive	2 (40.0)	0	2 (40.0)	1 (20.0)	
Triple negative	8 (40.0)	2 (10.0)	6 (30.0)	4 (20.0)	

*JBCS* Japanese Breast Cancer Society

## Discussion

To the best of our knowledge, the present study is the largest individual patient-based pooled analysis of the different definitions of pCR in breast cancer patients who were enrolled in prospective studies of neoadjuvant anthracycline–taxane-based chemotherapy in Japan. We first compared 5 definitions of pCR: QpCR, CpCR, CpCRbn, SpCR, and SpCRbn. By definition, SpCR is the most vigorous response in the breast, and SpCRbn represents the most complete response to NAC, and the order of pCR rates is theoretically as follows: QpCR ≥ CpCR ≥ SpCR, CpCR ≥ CpCRbn, SpCR ≥ SpCRbn, CpCRbn ≥ SpCRbn [3, 4]. In agreement with this, the order of pCR rates was QpCR > CpCR > CpCRbn > SpCR > SpCRbn in the present study. Similarly, in a meta-analysis of 12 neoadjuvant randomized trials conducted by the Collaborative Trials in Neoadjuvant Breast Cancer (CTNeoBC) (*n* = 13,125), pCR rates of CpCR, CpCRbn, and SpCRbn were 22, 18, and 13 %, respectively [15]. In addition, in the study by von Minckwitz et al. [6], the rates of ypT0/is/micypN0/+, CpCR, CpCRbn, and SpCRbn were 30.2, 22.8, 19.8, and 15.0 %, respectively. Thus, pCR rates could vary according to the definition, and this non-equivalency in the definition of pCR could be problematic when reviewing the results of NAC for approval under the accelerated approval regulations [8]. In this respect, the CTNeoBC has recommended SpCRbn or CpCRbn for the definition of pCR in consideration of the consistency, while von Minckwitz et al. have concluded that SpCRbn could best discriminate between patients with favorable and unfavorable outcomes [6, 15]. Unfortunately, as these meta-analyses included the studies performed in Europe and United States, it still remains uncertain whether these recommendations are applicable in Japan.

The present study found prognostic significance of CpCRbn in addition to QpCR and CpCR, and SpCR and SpCRbn were not significantly associated with prognosis. Thus, CpCRbn is considered to be the preferable definition of pCR. As for the prognostic significance of SpCR and SpCRbn, our results seem to contradict the previous findings described above [6, 15], and the prognostic significance of tumor size appears to be dependent on the definition of pCR. This observation might be attributable to a much lower number of patients with SpCR or SpCRbn than patients with QpCR, CpCR, or CpCRbn and a limited number of events, resulting in a much lower statistical power to show prognostic significance in the present study. Less intensive NAC might not the cause of lower SpCR or PpCRbn rates, as every patient received an anthracycline-containing regimen and docetaxel with acceptable compliance in this pooled analysis [10–12].

On the other hand, the prognostic significance of nodal status and subtype was consistent regardless of the definition of pCR. As for nodal status, this observation is consistent with other studies [6, 16, 17]. For example, the study by Bear et al. [16] has demonstrated that pathologic nodal status was a strong predictor of survival irrespective of pathologic response to the breast. As for subtype, the potential limitations of this study should be addressed; i.e., we could not divide luminal subtype into luminal A subtype and luminal B/Her2-negative subtype, and the sample size of patients with TN or Her2-positive tumors was small. Nevertheless, it is noteworthy that patients with TN tumors could achieve pCR, but TN tumors were associated with poor prognosis as compared to luminal tumors in the present study. This observation is in line with the study demonstrating that patients with TN tumors have increased pCR rates as compared to patients with non-TN tumors, and patients with pCR have excellent and comparable survival, while those with invasive RD have significantly worse survival if they have TN tumors versus non-TN tumors [17]. Thus, the current issue regarding TN tumors appears to be that high pCR rates obtained in patients with TN tumors do not necessarily have a meaningful effect on prognosis of the entire group of patients with TN tumors [17]. It is also interesting to note that the pCR rate was high in patients with Her2-positive or luminal/Her2-positive tumors as compared to patients with luminal tumors, but Her2 positivity had no prognostic significance in the present study. We did not use trastuzumab as a part of NAC and only 23 % of patients with luminal/Her2-positive or Her2-positive tumors received postoperative trastuzumab as reported previously [14]. As trastuzumab is now used routinely, however, it is possible that the prognostic gap between luminal/Her2-positive or Her2-positive tumors and luminal tumors could be wider today. In fact, several studies have demonstrated an influential effect on achieving pCR through inclusion of Her2-directed therapy with NAC as well as improvement of prognosis through adjuvant use of Her2-directed therapy [18, 19].

We also found that patients who achieved Grade 3 or Grade 2b had a more favorable prognosis than patients who did not. In this respect, it should be noted that invasive RD after NAC includes a broad range of actual responses from near pCR to frank resistance, and Grade 2b differs from the other studies including focal RD to pCR in the extent of RD [6, 14, 20, 21]. Grade 2b was strictly defined as only a few remaining isolated cancer cells, while the other studies considered up to 5 mm of RD as focal and found that focal invasive RD, ypTis, and ypN+ were associated with increased relapse risk [6]. In association with this, it is interesting to note that Symmans et al. [7] found minimal RD, i.e., residual cancer burden (RCB)-I had the same 5-year prognosis as patients with no RD. In that study, pathologic responses were subdivided into RCB-0 (ypstage0, no RD), RCB-1, RCB-II (moderate RD), and RCB-III (extensive RD) by calculating RCB as a continuous variable from the primary tumor dimensions, cellularity of the tumor bed, and the number and size of nodal metastases. Needless to say, the inclusion of RCB-1 or Grade 2b would expand the subset which could be identified as having benefited from NAC, and further study should clarify the biology of the remaining cancer cells observed in RCB-1 or Grade 2b.

Furthermore, we found a certain level of association between the first site of recurrence and pCR or subtype. In particular, neither bone nor brain was the first site of recurrence in patients with pCR, irrespective of the definition of pCR, and bone was not the first site of recurrence in patients with luminal/Her2-positive or Her2-positive tumors. As for soft tissue recurrence, the results of the present study are consistent with the study by Caudle et al. [22] demonstrating that Her2-positive and TN tumors were associated with higher rates of locoregional recurrence. Similarly, Liedtke et al. [17] reported that TN tumors had higher rates of recurrence in viscera and soft tissue and lower rates in bone. As for brain metastasis, Shimizu et al. [23] found that the brain was not the first site of recurrence in patients with luminal/Her2-positive or Her2-positive tumors who were not treated with trastuzumab, while it was the most common site of first metastasis in patients treated with trastuzumab as a part of NAC. Taken together, the first site of recurrence could vary according to pathologic response, subtype, and treatment. So far, limited data are available for the first site of recurrence after NAC, and whether intensive follow-up could improve survival has not yet been demonstrated. Further studies should examine the utility of the individualized surveillance based on the pathologic response, subtype, and treatment.

In conclusion, the prognostic significance of pCR as well as its rate varied according to the definition of pCR. Subtype and nodal status were prognostic variables independent of the definition of pCR. This study underscores the needs of standardization of the definition of pCR and provides supporting evidence to CTNeoBC.
